# Zinc homeostasis and redox alterations in obesity

**DOI:** 10.3389/fendo.2023.1273177

**Published:** 2024-01-08

**Authors:** Cristina Franco, Lorella Maria Teresa Canzoniero

**Affiliations:** Department of Science and Technology, University of Sannio, Benevento, Italy

**Keywords:** zinc status, oxidative stress, buffering, ZnT, ZIP, metallothioneins

## Abstract

Impairment of both cellular zinc and redox homeostasis is a feature of several chronic diseases, including obesity. A significant two-way interaction exists between redox metabolism and the relatively redox-inert zinc ion. Redox metabolism critically influences zinc homeostasis and controls its cellular availability for various cellular functions by regulating zinc exchange from/to zinc-binding proteins. Zinc can regulate redox metabolism and exhibits multiple pro-antioxidant properties. On the other hand, even minor disturbances in zinc status and zinc homeostasis affect systemic and cellular redox homeostasis. At the cellular level, zinc homeostasis is regulated by a multi-layered machinery consisting of zinc-binding molecules, zinc sensors, and two selective families of zinc transporters, the Zinc Transporter (ZnT) and Zrt, Irt-like protein (ZIP). In the present review, we summarize the current state of knowledge on the role of the mutual interaction between zinc and redox homeostasis in physiology and pathophysiology, pointing to the role of zinc in the alterations responsible for redox stress in obesity. Since zinc transporters primarily control zinc homeostasis, we describe how changes in the expression and activity of these zinc-regulating proteins are associated with obesity.

## 1 Introduction

It has been widely documented that oxidative stress (OS) occurs in overweight and obesity and plays a central role in obesity-related comorbidities (1). Several conditions underlie OS in obesity, including hyperglycemia, hyperlipidemia, chronic inflammation, and inadequate antioxidant defenses, which are closely linked, although some contribute more than others.

Impairment in antioxidant defenses in obesity has also been associated with a deficiency in various microelements and vitamins. A combined vitamin and trace element deficiency has been demonstrated in overweight individuals, which worsens with increasing obesity (2). In fact, obese individuals frequently experience low levels of carotenoids, vitamins A, B6, C, D, and E (3, 4), as well as deficiency in selenium, magnesium, iron, and zinc microelements (4, 5).

Here we review the current state of knowledge on the interplay between zinc and redox homeostasis in obesity. Since zinc transporters primarily control zinc homeostasis, we also describe how changes in the expression and activity of these zinc-regulating proteins are associated with obesity.

## 2 Redox signaling and homeostasis: two faced role in obesity

OS relies on the excessive production of Reactive Oxygen Species (ROS) and Reactive Nitrogen Species (RNS) (RO(N)S) (6). Recently the concept of RO(N)S as harmful molecules has been reconsidered. It is now known that ROS are not always “evil” and antioxidants are not always “good,” but that the extent and contest in which ROS are produced determine whether it can cause beneficial or harmful effects on living systems. This “two-faced” nature of RO(N)S is supported by their functional role as signaling molecules (second messengers) in numerous redox-regulated biological processes such as cell division, differentiation, death, host defense, and metabolic regulation (7). RO(N)S are an integral part of normal cell signaling, responsible for reversible redox-based (oxidation/reduction) post-translational modifications (redox PTMs) of reactive and redox-sensitive sulfur-containing amino acid residues of cell signaling pathway components in a highly selective manner (7). Hence, as with other signaling molecules, the generation of RO(N)S is regulated to avoid RO(N)S concentrations higher than those tolerated by cells (8). Paradoxically, they also act as sensors of changes in the cellular redox state and contribute to maintain redox homeostasis (9).

In this regard, the close relationship between ROS and glucose-lipid metabolism is not surprising, considering that the cell’s redox status is related to glucose and lipid metabolism (10, 11). However, the production of ROS is not only the consequence of glucose and lipid usage, but it also controls glucose and lipid metabolism. Indeed, a close relationship exists between ROS and insulin signaling in its target cells (12, 13). Following cellular insulin stimulation, a transient burst of hydrogen peroxide (H_2_O_2_) is essential for fine-tuning insulin signal transduction (14, 15). Namely, H_2_O_2_ can trigger biochemical modifications through oxidation of the reduced cysteine thiol side chains of protein tyrosine phosphatases (PTPs) that negatively regulate insulin signal transduction, leading to their inactivation (14, 16). In addition, ROS promotes glucose uptake by positively regulating gene expression of genes encoding glucose transporters (GLUTs) and signaling pathways responsible for translocating GLUTs from intracellular vesicles to the plasma membrane (10).

In contrast, chronic malnutrition and consumption of high-fat and high-carbohydrate meals deliver an excessive amount of energy substrates to the metabolic pathway in adipose and non-adipose cells, which, in turn, can increase the production of ROS, mainly via the mitochondrial electron transport chain (17). Greater availability of reducing equivalents from increased fatty acid and glucose loading results in less efficient oxidative phosphorylation in mitochondria that yields relatively large amounts of superoxide anion (O_2_^-·^). Impaired glucose utilization favors the occurrence of hyperglycemia, which enhances OS by oxidative degradation of glucose through autoxidation processes (18). Similarly, accumulated lipids are themselves targets of oxidation, causing an increase in lipid peroxidation (19). Lipid peroxidation products in turn can be released from adipose tissue and enter the liver, where they can alter the respiratory chain, forming more ROS and starting a vicious cycle (20).

Concomitantly, expanded adipose tissue synthesizes and secretes huge amounts of cytokines and chemokines, collectively defined adipochemokines or adipokines, such as leptin, that promote infiltration of adipose tissue by macrophages with subsequent overproduction of RO(N)S and inflammatory cytokines, leading to adipose tissue inflammation (21, 22). Such a process is triggered by OS, which leads to the activation of transcription factors that control the expression of proinflammatory cytokines, which further increase RO(N)S production (23).

Finally, the lack of antioxidant defenses, both in the form of enzymatic and nonenzymatic molecules, can contribute to OS in obesity. Indeed, reduced expression of superoxide dismutase (SOD), catalase, and glutathione peroxidase (GPX) enzymes has been described in obesity (19). It is conceivable that reduced activity of nuclear factor E2-related factor 2 (Nrf2) that controls the expression of diverse antioxidant enzymes contributes to weakening enzymatic antioxidant defenses in insulin resistance and obesity, thereby worsening OS (24).

Several studies have addressed the relationship between zinc status and the changes in obesity in animal models and obese individuals (25). Interestingly, zinc participates in all the primary metabolic processes contributing to OS in obesity (1). Although zinc is relatively redox-inert in living organisms, it can regulate redox metabolism and exhibits several pro-antioxidant properties (26). Similarly, redox metabolism regulates zinc exchange from/to zinc-binding proteins and controls zinc availability for various cellular functions. This two-way interaction between redox metabolism and zinc ion entails that even small perturbations in zinc status and zinc homeostasis can affect cellular and systemic redox homeostasis (27). Hence, it is conceivable that changes in dietary zinc status may exacerbate OS in obesity.

## 3 Zinc: an essential micronutrient

**Figure 1:**
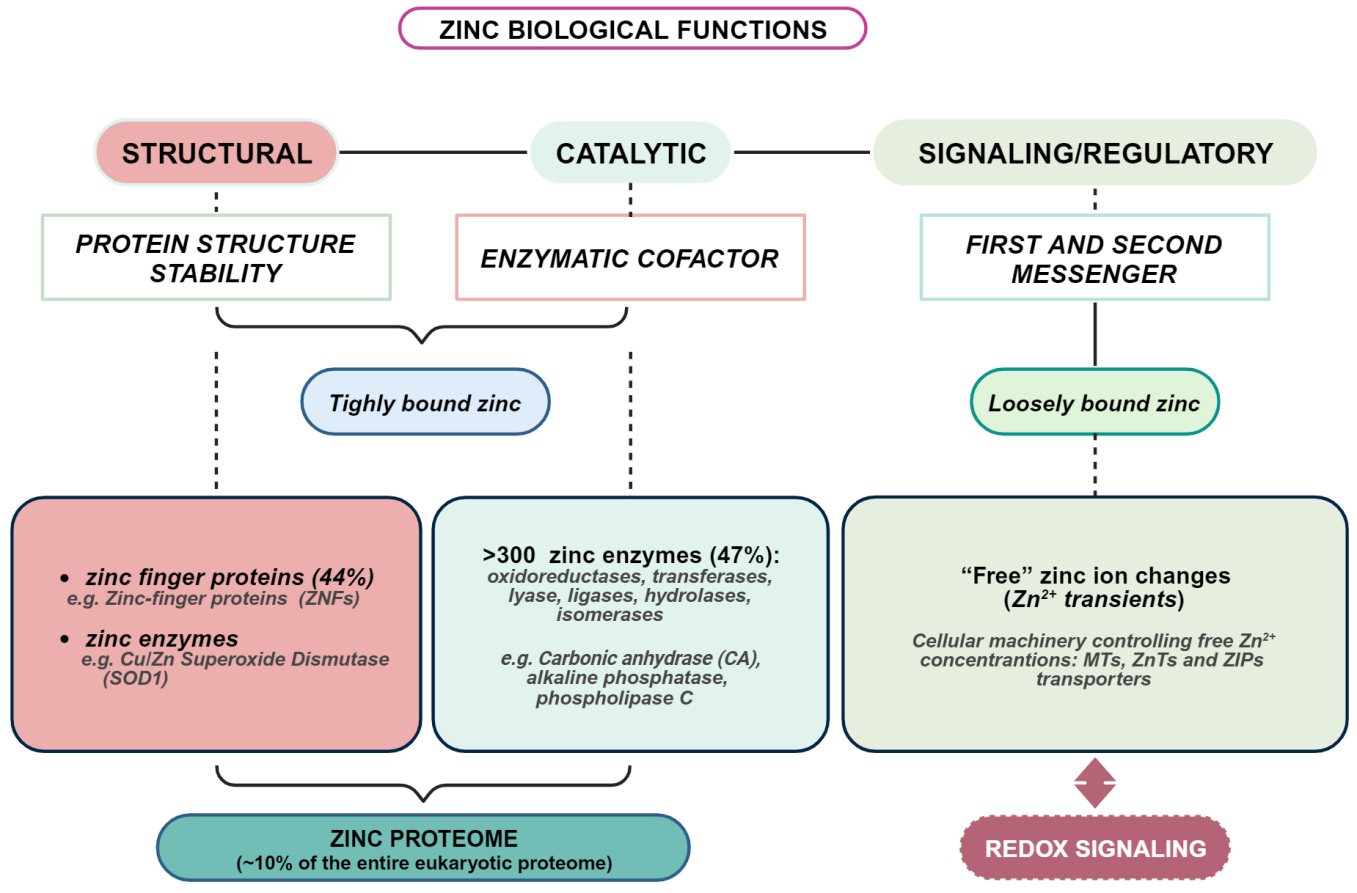
Overview of biological functions of zinc in living organisms. Created with BioRender.com.

The importance of zinc to human health has been increasingly appreciated since the first evidence by Prasad (28), and much has been learned about the molecular basis of its indispensability. However, many aspects of zinc biology remain to be further explored. Zinc is the second most abundant transition metal in living organisms after iron and the most abundant intracellular metal. Albeit zinc is considered a dietary microelement, intracellularly, it reaches very high concentrations (29). Indeed, of all total 2-3 g of body zinc, only 0.1% is present in the plasma, most of which is bound to proteins, whereas the remaining 99.9% is confined within cells (30). In plasma, zinc is mainly bound to albumin, α-macroglobulin, and transferrin (31–33), while only a very small fraction, less than 2%, is present as free zinc (33). It is present throughout the body, especially in skeletal muscle (~60%) and bone (~30%), followed by skin and liver. The remaining fraction is distributed among the other tissues and organs, including prostate, pancreas, heart, kidney, and brain (34, 35). Zinc is critically involved in cell proliferation, differentiation, survival, apoptosis, and neurotransmission (36, 37). Estimates place overall intracellular zinc concentration between 200-300 μM, depending on cell type (38). Such high intracellular concentration is substantiated by the fact that a huge number of proteins requires zinc: about 1 in 10 proteins (~3000 proteins) contains a zinc-binding motif (39). In about 90% of the overall zinc-dependent proteins, zinc is required as a catalytic cofactor by 300 enzymes of all classes and a structural component of thousands of protein domains, such as the “zinc finger” domain of numerous transcription factors (35). Such a large number of proteins that require zinc to fulfill their function stimulates reflection on how complex the entire zinc proteome is and how sophisticated is the mechanism by which proteins gain access to zinc ions within a cell. Maintaining cellular zinc levels within an appropriate range is critical, as even a slight deficiency or excess can significantly affect human growth, health, and well-being (40, 41) (**Figure 1**).

The average daily zinc intake (Recommended Dietary Allowance, RDA) sufficient to meet the nutrient requirements of healthy adults is 11 mg for men and 8 mg for women. Depending on age, children require 2-8 mg for both sexes (42). The tolerable upper limit (UL) for zinc has been set at 40 mg/day (43). Zinc is naturally present in a wide variety of foods. The major dietary sources of zinc are shellfish (oysters, crabs, lobsters), red meat, poultry, pork, eggs, dairy products, legumes, nuts, seeds, whole grains, and vegetables (44, 45). However, zinc from plant sources is less bioavailable than from animal foods because they contain large amounts of phytic acid and some other indigestible zinc-binding ligands that make zinc unavailable for absorption (45–49).

Unfortunately, zinc deficiency in humans remains a significant global public health problem. In low- and middle-income countries, an estimated 4% of childhood morbidity and mortality is due to severe zinc deficiency (50, 51). However, slightly inadequate zinc intake is also observed in young children and the elderly over 69 years of age in developed countries (42, 52), suggesting that many factors interact to cause zinc deficiency (53). In particular, dietary habits and food preferences, such as high consumption of phytate-containing foods, e.g., corn, cereals, rice, and legumes, reduce zinc absorption (48). In addition, chronic diseases such as diabetes, gastrointestinal, liver, and kidney diseases, or infections are considered important risk factors for zinc deficiency (54–56).

The body responds to insufficient intake with rapid metabolic adaptations aimed at reducing endogenous losses, primarily by lowering zinc-dependent processes, such as growth and immune system functions. In fact, zinc deficiency mainly affects the immune, skeletal, gastrointestinal, epidermal, nervous, and reproductive systems (49, 57, 58). As a result, severe zinc deficiency leads to serious complications such as immune system dysfunction with recurrent infections, growth retardation, weight loss, alopecia, diarrhea, dermatitis, hypogonadism, hematologic abnormalities, mental disorders, and increased oxidative stress (55, 59–61). However, mild/moderate zinc deficiency is more common than severe zinc deficiency and, in humans, is usually associated with growth retardation, male hypogonadism, skin changes, loss of appetite, loss of taste, mild weight loss, mental lethargy, abnormal dark adaptation, and delayed wound healing (59, 62). Therefore, an adequate supply of zinc is critical for both nutritional status and treatment of various diseases. In this regard, zinc supplementation is potentially beneficial and may serve to correct intercurrent deficiency, such as in preterm infants (63), or as adjunct therapy for various acute and chronic diseases (59, 64).

However, zinc deficiency is not the only health problem associated with zinc intake. Although in healthy individuals the risk of zinc accumulation is much lower than that of zinc deficiency and is a relatively rare event, long-term exposure to elevated zinc concentrations well above the UL results in clinical manifestations of zinc toxicity (65, 66). Zinc toxicity mainly results from dietary supplements, including multivitamins, or the overuse of denture adhesive creams (67). Conversely, even if they contain high amounts of the mineral, excess zinc from food sources alone is relatively harmless. In this context, healthy infants are often unnecessarily exposed to the risk of zinc poisoning, primarily through consumption of supplements, foods, and beverages used to meet needs during growth and to prevent possible inadequate intakes in early childhood (68). Adverse effects of high zinc intake include nausea, dizziness, headache, upset stomach, vomiting, loss of appetite, and lower immunity (65). It is also known that intake of very high doses of zinc supplements can impair the HDL: LDL ratio and copper absorption, resulting in decreased serum levels of HDL and copper, respectively (69–71).

In addition, zinc status in humans and other organisms is of particular importance for the optimal maintenance of a normal gut microbiota composition (72). Zinc shapes host–pathogen interactions and actively protects the host from pathogen invasion. In addition, zinc contributes to maintaining the integrity of the intestinal barrier (73) by regulating the activity of alkaline phosphatase (74). On the other hand, competition for zinc between the immune system and the pathogen and zinc accumulation in immune cells represents the first line of defense against host colonization to limit bacterial infection, a process known as nutritional immunity (29, 74). On the other hand, zinc also serves as an essential micronutrient in prokaryotic cells (29) and is involved in many aspects of their biology. Therefore, it is not surprising that both zinc deficiency and excess can significantly affect the gut microbiota by altering microbial diversity and changing susceptibility to bacterial infections (68, 75–77). This aspect is of great interest considering that dysbiosis of the gut microbiome is causally associated with obesity in humans (78–80).

Even in severe deficiency, a slight increase in dietary zinc intake can rapidly improve clinical symptoms and restore the small amount of zinc loss. An effective homeostatic mechanism prevents variations in tissue zinc and meets tissue requirements over a wide range of zinc intakes (81). The daily zinc requirement is relatively low due to the slow turnover of body zinc (1/1000 per day); however, a correct daily dietary intake is essential because it influences the size of a small but significant endogenous zinc pool, the so-called “exchangeable zinc pool” (EZP), which is probably the most important zinc reservoir (61). EZP accounts for approximately 10% of total body zinc (51) and is localized mainly in plasma and liver (82, 83). The metal ion that makes up this pool is readily mobilized and exchanged with tissues, making zinc available for all functions that require this metal (84).

Consequently, an increase in tissue uptake could decrease serum zinc if it is not rapidly absorbed, transferred to plasma, and replaced within the EZP. The availability of zinc controls the rate of EZP turnover (81). EZP turnover increases markedly (~35) when zinc intake is deficient (85) and immediately decreases when zinc intake is restored (85). Interestingly, intake of glucose and fat results in a significant decrease in plasma zinc, albeit slowest with fat meals, likely due to the slower absorption rate (81). Most importantly, the extent of the decline in plasma zinc induced by glucose and fat depends on the energy intake rather than the meal composition (81, 86).

## 4 Zinc status and obesity

The propensity to develop obesity varies between the sexes. Indeed, obesity is a sexually dimorphic trait and is more common in boys than in girls (6% of girls and 8% of boys). Conversely, it is more frequent in adult women than in age- and weight-matched men (World Health Organization. Obesity and overweight. Available at: https://www.who.int/news-room/fact-sheets/detail/obesity-and-overweight). Similarly, it is generally observed that women, both normal weight and obese, have slightly higher serum zinc levels than men, although not significantly (87, 88). Nevertheless, changes in zinc status occur in obese children and adults regardless of gender. In particular, obese patients of both sexes frequently exhibit alterations in the metabolism of this trace element, as demonstrated by the significantly lower serum zinc concentrations that they exhibit compared with lean control subjects (87–101). In the same individuals, zinc balance is worsened by increased hyperzincuria due to increased urinary zinc excretion (87). The latter aspect contrasts sharply with conditions characterized by dietary zinc deficiency, in which the body attempts to conserve zinc and reduce its excretion (102).

In addition, hypozincemia in obese individuals is negatively associated with anthropometric parameters such as body mass index (BMI) and waist circumference, as well as with biochemical parameters such as fasting blood glucose, insulin and leptin (88, 96, 98, 103, 104). Also, women with poorer zinc status had higher body weight, waist circumference, and plasma glucose levels than women with normal levels of this trace mineral (101). The association between zinc and obesity has been underscored in genetically obese (ob/ob) mice that exhibited hypozincemia and hyperzincuria (95) and in null mice for zinc-binding proteins metallothioneins (MTs) that spontaneously develop obesity (105).

Alterations of zinc distribution occur in obese mice. For instance, zinc levels were significantly lower in the pancreas, whereas higher levels were found in muscle, brown and white adipose tissue, and liver (106, 107). Alterations in the sequestration and zinc transport mechanisms in the liver and adipose tissue may be responsible for the hypozincemia observed in obesity (106). Such a decrease in serum zinc would affect the zinc supply to all tissues, reducing the amounts needed to maintain EZP.

However, even though hypozincemia seems to be a common feature of obesity, it is not a direct cause of the disease (108). No significant differences in body weight, adipose tissue mass, or fat distribution are observed in obese individuals on a low zinc diet. In contrast, even modest weight loss in obese individuals following a hypocaloric diet increased circulating zinc to normal levels (89, 101, 109). Most important, obesity-induced hypozincemia is inversely correlated with OS markers (59). Weight loss following moderate caloric restriction while restoring normal zinc levels improved obesity-related OS (110).

## 5 Zinc and obesity-related metabolic abnormalities

Numerous observations emphasize the role of nutritional zinc in all the significant features of obesity. From a mechanistic point of view, the biological functions of zinc are closely associated with all the major metabolic mechanisms that generate OS in obesity, especially with a lack of antioxidant defenses, but also with insulin resistance, hyperleptinemia, and chronic low-grade inflammation (1, 111).

### 5.1 Zinc and obesity-related insulin resistance and hyperleptinemia

Zinc has insulin-mimetic activity and is involved in the synthesis, storage, and release of insulin in pancreatic β-cells (112). In particular, zinc is stoichiometrically associated with insulin molecules in the secretory granules of pancreatic β-cells to maintain the crystalline structure of insulin and prevent its degradation by the action of proteolytic enzymes (113, 114). Furthermore, upon glucose stimulation, zinc is released along with insulin into the extracellular space, exerting autocrine and paracrine effects within the pancreas (113). It controls insulin secretion by positively regulating the ATP-activated K^+^ channel (KATP) activity and potentiating KATP currents, ultimately reducing cellular excitability and, thus, insulin release (115). Moreover, zinc promotes the action of insulin (116, 117) by lowering blood glucose levels and sensitizing target cells in muscle, liver, and adipose tissue to insulin signaling and exerting a regulatory function on various signaling cascades (118–122). Specifically, changes in intracellular zinc appear to be crucial in modulating insulin response through crosstalk with phosphorylation signaling pathways. The mechanisms underlying these effects include the inhibition of protein tyrosine phosphatase 1B (PTP1B), which is physiologically responsible for insulin receptor inhibition through dephosphorylation and attenuates insulin signal transduction (123, 124).

Resistance to the cellular effects of insulin is a significant contributor to obesity (125) and gradually leads to a failure of pancreatic β-cell function, which inevitably results in impaired insulin secretion due to a progressive loss of insulin sensitivity of peripheral target tissues. Initially, pancreatic β-cells attempt to compensate for decreased sensitivity to insulin action by increasing the rate of insulin synthesis and release through hypertrophic and hyperplastic processes in pancreatic β-islets to improve glucose sensing until the compensatory potential of the pancreas is exhausted (126, 127).

Zinc deficiency has been shown to decrease the total number of insulin granules in pancreatic β-cells and to impair insulin sensitivity and glucose tolerance in obese rats and mice (128, 129). As in rodents, obese individuals with lower dietary zinc intakes have higher insulin levels than obese ones with normal dietary zinc intakes (97). In *ob/ob* mice, zinc supplementation markedly attenuates glucose-induced insulin secretion and lessens fasting plasma glucose levels (107). Similarly, zinc treatment leads to a change in metabolic profile with beneficial effects on insulin sensitivity in both obese children and adults (130–134).

Zinc can improve glucose utilization in insulin-resistant individuals through its insulin-like effects, increasing glucose uptake in insulin-resistant muscle cells by upregulating critical components of the insulin signaling cascade (122). These results are also supported by the blood glucose-lowering effects of zinc compounds observed in several preclinical studies (135–138). Concerning the potential risk of insulin resistance resulting from impaired zinc status in obesity, Cruz and his coworkers (131) systematically reviewed the results of several clinical trials conducted to determine the efficacy or inefficacy of zinc supplementation in obese men and women. The available evidence supports the notion that improving zinc status helps relieve insulin resistance (131, 139).

Similarly, zinc’s regulation of leptin production has been a matter of intense research. Leptin functions as a hormone to give information about the status of body fat depots by acting on the hypothalamus to diminish food intake and increase energy expenditure to maintain constant adipose tissue mass. Brain leptin controls the hypothalamic production of a central appetite-regulating neuropeptide called neuropeptide Y (NPY), which, in turn, stimulates appetite (140, 141). During fasting, NPY levels are high while leptin levels are low, stimulating appetite. It is now apparent that, among other biological functions, zinc regulates appetite, modulating leptin production (55, 104, 142, 143). Interestingly, in healthy humans and rodents, dietary zinc deficiency decreased circulating leptin levels (104, 143–145), whereas zinc supplementation increased leptin levels proportionally to zinc adjustments (104). Thus, in non-obese individuals, zinc provides a regulatory signal for food intake via leptin that can explain the decreased appetite and anorexia observed in zinc deficiency (105,145. 144). Thus, in healthy individuals, zinc deficiency caused a general reduction of NPY, which may ultimately lead to weight loss and anorexia (146, 147).

Consequently, changes in the leptin-NPY axis may occur in obesity, but paradoxically, the vast majority of obese individuals do not have reduced but higher circulating leptin concentrations that do not lead to a reduction in appetite, indicating leptin resistance (148, 149). In obesity, a low zinc diet alters leptin production, although in this case zinc deficiency leads to increased rather than decreased serum leptin levels. Indeed, high blood and adipose leptin levels are associated with low serum zinc levels in obese mice (108, 150). As in mice, an inverse association between dietary zinc intake and leptinemia has also been observed in young obese women (151). Zinc deficiency may also exacerbate hyperleptinemia in obesity by other mechanisms. In this context, as in the case of insulin, PTP1B directly regulated leptin by controlling the phosphorylation state of the leptin receptor and negatively affecting leptin sensitivity (152). Zinc deficiency could contribute to leptin insensitivity in non-adipose tissues such as liver and muscle through loss of the inhibitory effect of zinc on PTP1B. In addition, a positive association between hyperinsulinemia and hyperleptinemia in obesity has been observed. Specifically, higher blood leptin levels reflect higher insulin resistance (153). Mechanistically, chronic hyperinsulinemia resulting from decreased responsiveness to insulin promotes excessive synthesis and release of leptin from adipose tissue (91, 154).

### 5.2 Zinc and obesity-related inflammation

Obesity is an inflammatory disease (155). Indeed, inflammation begins in adipose tissue and gradually spreads to adjacent sites such as skeletal muscle and liver until a systemic, low-grade inflammatory state, also known as metabolic inflammation, develops and fails to resolve (156). Adipose tissue is able to synthesize and release locally and systemically pro-inflammatory and anti-inflammatory cytokines, such as leptin but also TNF-α, IL-6, adiponectin, and resistin. All fat depots are composed of adipocytes, resident innate and adaptive immune cells, represented mainly by macrophages, but also including neutrophils, dendritic cells, eosinophils, natural killer cells, innate lymphoid cells (ILCs), and B and T cells distributed in the stromal vascular fraction surrounded by a dense blood network (157). Excessive fat accumulation inflames adipose tissue during obesity and favors adipose tissue macrophages (ATM) polarization from an anti-inflammatory to a pro-inflammatory phenotype, leading to further recruitment and infiltration of macrophages and other immune cells. The latter, in turn, massively produce and release pro-inflammatory cytokines, leading to an inflammatory cycle that exacerbates adipose tissue inflammation and triggers a systemic inflammatory cascade through immune cell infiltration of other insulin-dependent tissues (22).

In this regard, the dual function exhibited by leptin, namely as hormone and cytokine, is paradigmatic of the close and evolutionary conserved tie between the metabolic and immune system. Leptin acts as a modulator of the innate and adaptive immune response and has immunological activity functioning as cytokine (158–160). As such, leptin, like many immune system mediators, functions as signaling molecules of both the immune and metabolic systems and is considered the cornerstone signal that links these two systems, as through which they regulate and influence each other (158). In fact, leptin acts as a chemoattractant for monocytes/macrophages and is required for macrophage activation and cytokines expression of TNF-α, IL-6, and IL-12 (161). It derives that, in obesity, hyperleptinemia may therefore contribute to macrophage accumulation in tissues (162–164). An adequate dietary zinc intake is pivotal in the maintenance of the delicate balance between inflammatory and metabolic responses. In fact, in zinc deficient-obesity, leptin rise promoted the increase in macrophage infiltration in adipose tissue of mice fed a high-fat diet (108).

Yet, the influence of zinc on the immune response is not limited to its regulatory role on leptin levels but refers to the specific role that zinc plays in immunity compared to all other microelements. Indeed, zinc availability supports and influences both humoral and cell-mediated immune responses, affects many aspects of the immune system, and contributes to the maintenance of immune system functions, including the inflammatory response (165–168). Accordingly, both severe and mild zinc deficiency can weaken the immune system (61). More interesting is the fact that zinc is an anti-inflammatory agent that is able to reduce cytokine production (169) and zinc deficiency is not only associated with immune deficiencies but can also cause systemic inflammation (97). Therefore, zinc deficiency may directly contribute to the inflammatory state of adipose tissue in obese individuals, and it may also affect the amount of leptin produced and released by this tissue which ultimately has a proinflammatory effect itself and promotes the secretion of other cytokines. Accordingly, obese subjects with a lower zinc status displayed a sustained inflammation and activation of the immune response (97). Likewise, lower dietary zinc intake negatively associates with levels of IL-6 and leptin in young obese women with respect to normal zinc intake counterparts (151). Importantly, sex-related differences in the inverse relationship between zinc status and inflammatory markers have been found (170), which, in turn, may influence susceptibility to the development of diseases, particularly in women. Regarding the effects of zinc on obesity-related inflammation and its complications, zinc supplementation combined or not with restricted caloric diets has showed to modify inflammatory markers, reducing the levels of IL-6 and C-reactive protein (CRP) in obese individuals (100, 151). Of note, CRP levels are directly related to those of IL-6 (171), and IL-6 together with TNF-α concurs to reduce the sensitivity to insulin action (172, 173). While experimental and clinical data consistently indicate that long-term zinc supplementation directly improves inflammatory markers (100), clinical studies showed conflicting results on the effects of zinc on anthropometric measurements (e.g., weight, BMI, waist and hip circumference) (143, 150, 174, 175). Of relevance, the improvement in the inflammatory process observed following zinc supplementation is comparable to that obtained in case of body weight reduction. Moreover, weight loss alone produces the normalization of reduced zinc levels (109).

Cytokines themselves may, at least in part, cause the redistribution of zinc under inflammatory conditions (176). Remarkably, IL-6 is able to regulate metallothionein expression (177). Even more interestingly a polymorphism in the gene encoding metallothionein 2A (MT2A) is associated with higher plasma levels of IL-6, hyperglycemia, and marked zinc deficiency in patients with the AA genotype than in carriers of the AG allele, suggesting the presence of a specific genetic background that may influence susceptibility to the development of zinc deficiency associated with inflammation (177).

## 6 Zinc homeostasis

Eukaryotic cells have evolved a complex machinery consisting of importer/exporter proteins, sensors, and MTs to ensure that free zinc ion levels are in the femto/pico-molar range.

However, even in a buffered environment such as the cytoplasm, short-term intracellular increases in free zinc occur, typically in a narrow concentration range from picomolar to low nanomolar, referred to as “zinc transients” or “zinc signals”. In contrast to structural and catalytic functions, such zinc changes are due to a very small intracellular zinc pool that is not stably bound to proteins and is therefore referred to as ‘labile’ or “rapidly exchangeable”. It can be transiently released within and from cells (178), allowing zinc to exert regulatory functions and act as a first and second messenger.

Zinc signals originate from extracellular zinc translocation and intracellular release of zinc from intracellular stores or MTs (116). When zinc is released into the surrounding milieu, it can be taken up by neighboring cells through transport mechanisms responsible for translocating zinc into the cell (179). Extracellularly, zinc concentrations are extremely low, and their increase is probably due to the release of zinc stored in membrane-enclosed vesicles and released by exocytosis. Well known examples are the zinc released with glutamate at the synaptic level in the central nervous system (180) and zinc delivered with insulin to secretory vesicles released by pancreatic β-cells in response to stimulation with glucose (181).

Alternatively, zinc signals may originate from ions stored intracellularly. Similar to calcium, eukaryotic cells store zinc ions in some cellular compartments where they perform important functions, and they can be released in response to various stimuli (181–186). Remarkably, extracellular and intracellular zinc signals trigger a zinc burst, but the time scale of zinc release from intracellular stores is slightly slower than that of extracellular zinc flux (116, 187).

Finally, cellular zinc signals may result from its oxidative release from the thiol groups of MTs. In humans, at least 11 functional isoforms of MTs are known, divided into four classes (MT-1 to MT-4), all of which contain twenty conserved cysteine residues that give them the ability to coordinate seven zinc ions (188, 189). The unique intramolecular arrangement by which zinc ions are coordinated in MTs gives them the ability to bind zinc tightly but at the same time mobilize it readily without altering its valence. Indeed, in biological systems, the zinc ion is relatively redox-inert, exhibiting only one valence (Zn^2+^). In contrast, the thiol groups of the cysteine residues of MTs can be oxidized and reduced, which confers redox activity to the zinc clusters. Once oxidized or reduced, the thiol groups release or bind zinc (188). Because of the coupling of zinc binding/release with the ionization state of thiol groups of MTs display two major functions, namely zinc acceptor and zinc donor (190, 191). Certainly, such coupling links cellular zinc to the cellular redox state, as a shift to more oxidizing conditions results in the release of zinc, whereas a shift to more reducing conditions results in its binding (192). This is particularly interesting as there is growing evidence that redox signaling is critical for various cellular functions (6, 193).

The ionization state of MTs can be altered by cellular oxidants such as glutathione disulfide (GSSG) or ROS. Oxidation of the thiol groups causes the release of zinc from its binding sites on the MTs and the formation of disulfide bonds that can be reduced by glutathione (GSH). In contrast, cysteine-sulfur reduction leads to binding of zinc to MTs. Zinc released by MTs promotes further expression of MTs via the transcription factor Metal Regulatory Transcription Factor 1 (MTF1), which acts as a sensor of labile zinc levels (194, 195), to reduce zinc availability.

### 6.1 Pro-antioxidant actions of zinc

Although intimate relationship between zinc release from MTs and cellular redox state, zinc cannot be considered an antioxidant because it does not interact directly with oxidants to scavenge them. In contrast, it exerts this effect indirectly by interfering with or suppressing oxidative reactions and protecting cellular components from oxidative damage. Therefore, it should be referred to zinc as a pro-antioxidant (196).

**Figure 2:**
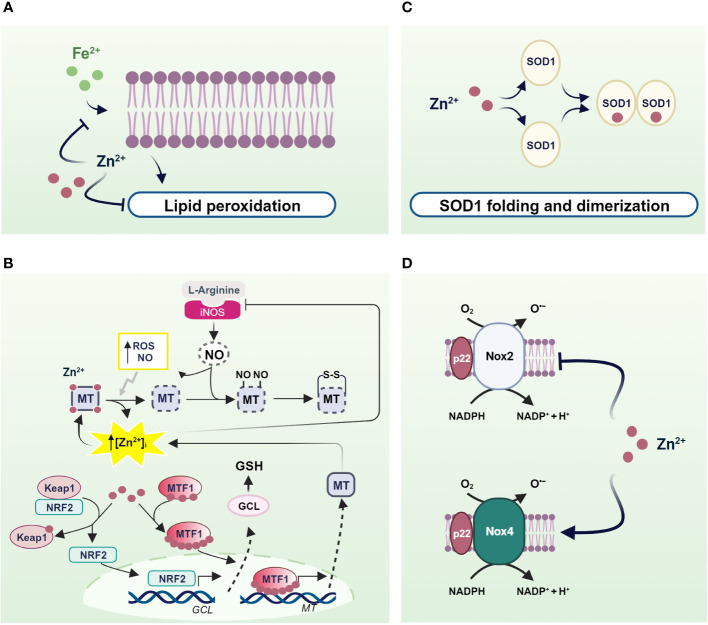
Mechanisms of pro-antioxidant actions of zinc in living organisms. **(A)** Zinc stabilizes the cell membrane by competing with redox-active metals and preventing the formation of highly oxidant lipid peroxides; **(B)** NO- and ROS-induced zinc release from MTs helps to counteract OS through the translocation of MTF1 and NRF2 to the nucleus, which in turn activate the transcription of genes encoding MTs and antioxidant defenses; **(C)** SOD1 and **(D)** NOXs as examples of enzymes in which zinc is an essential structural component and a regulator of the activity of ROS-producing enzymes. Created with BioRender.com.

Zinc acts as a pro-antioxidant with multiple actions integrated into the cellular system to defend against oxidants (197). First, zinc generally binds the negative charge of cell membrane phospholipids and, together with nonenzymatic antioxidants, protects membrane lipids from peroxidative damage caused by heavy metals. In particular, it competes with redox-active metals such as iron and copper, preventing the formation of highly oxidant lipid peroxides (198–200) (**Figure 2A**).

Besides to reduced free zinc levels, zinc binding to MTs directly protects the sulfhydryl groups of MTs from oxidation. In addition, the oxidation of MT causes zinc release and is coupled to MTs expression to confer protection against OS. In fact, zinc release from MTs contributes to counteract OS inducing MT expression by activating MTF1. A similar mechanism is observed after MTs thiol oxidation by nitric oxide (NO), H_2_O_2_, and GSSG (201–204) (**Figure 2B**).

Similarly, zinc released by MTs is capable of modulating the expression of antioxidant enzymes under the control of other zinc-modulated transcription factors. Interestingly, Nrf2 is emerging as an important regulator of cellular resistance to oxidants and OS (205). Under basal conditions, Nrf2 signaling is suppressed as it is sequestered in the cytosol by interacting with zinc metalloprotein Keap1 (Kelch-like erythroid cell-derived protein with CNC homology-associated protein 1) (206), which promotes its degradation via a ubiquitination proteasome system. As a result of various stimuli, the Nrf2/Keap1 complex can be dissociated and Nrf2 is free to migrate to the nucleus (207). In both healthy and stressed cells, released zinc from MTs can alter the conformation of Keap1 by binding to cysteine residues on Keap1, thereby reducing its affinity for Nrf2. As a result, Nrf2 migrates to the nucleus where it promotes the expression of genes involved in antioxidant defense (208) (**Figure 2B**).

In addition to MTs, zinc can be bound by other cellular components containing cysteine residues such as GSH (209). As with MTs, zinc binding to thiol groups protects them from oxidation. Furthermore, zinc/GSH interaction confers to the cell the capability to counteract redox changes, controlling zinc release from MTs. In fact, GSH binding to MTs provokes an increase in labile zinc levels affecting MT conformation and displacing zinc from its binding sites (210). In turn, zinc positively modulates the *de novo* synthesis of GSH through induction of Nrf2 transcription factor, which is responsible for the upregulation of the gene coding for the rate-limiting enzyme of GSH synthesis, glutamate cysteine ligase (GCL) (211). In addition, zinc stabilizes GCL preventing its cleavage by caspase 3 (212, 213) (**Figure 2B**).

Similarly, zinc exerts pro-antioxidant actions also as a cofactor of antioxidant enzymes. A well-known example of a zinc metalloenzyme is the copper/zinc superoxide dismutase (Cu/Zn SOD, SOD1), which rids the cells from superoxide radicals turning it into water and H_2_O_2_. Notably, zinc binding is needed for the proper functioning of SOD1. In fact, zinc stabilizes the native structure of each SOD1 monomer, accelerating its folding and promoting its dimerization. In contrast, zinc mis-metalation alters SOD1 folding and indirectly affects its catalytic activities (214) (**Figure 2C**).

There is convincing evidence that zinc also controls the expression and activity of NADPH oxidase (Nox), a superoxide-producing enzyme. Seven Nox catalytic components have been identified, namely Nox 1-5, Duox1, and Duox2. Interestingly, Nox 1, 2, 3, and 5 produce superoxide anions, whereas Nox4, Duox1, and 2 mainly generate hydrogen peroxide and release it into the extracellular space. However, the superoxide anion is rapidly disproportionated to the more stable product H_2_O_2_ by SOD. Moreover, unlike other members of the Nox family, Nox4 is constitutively activated, suggesting that it is actively involved in generating ROS, which plays a second messenger role in numerous physiological and biochemical processes (215). Interestingly, several lines of evidence suggest that Nox2- and Nox4-containing NADPH oxidase are differentially regulated by zinc. Specifically, intracellular zinc exerted an inhibitory effect on Nox2, as demonstrated by increased expression, activation, and activity of Nox2 under zinc deficiency, which was greatly attenuated by zinc enrichment (216). Accordingly, silencing of Nox2 attenuates the OS observed under zinc deficiency, suggesting that Nox2 is an essential regulator of OS under reduced zinc availability due to its ability to produce superoxide. Of note, by contrast, Nox4 is downregulated in zinc deficiency (216). This evidence is undoubtedly important considering the endogenous mechanism of coupling oxidative signaling via Nox4 and the insulin signaling cascade (15). In this context, the downregulation of Nox4 observed in zinc deficiency could promote depression of the insulin cascade (**Figure 2D**).

Moreover, zinc is not only a critical structural component of all isoforms of nitric oxide synthase (NOS) (217, 218) but also a key player in regulating both their expression and activity. More deeply, the ability of zinc to inhibit the production of NO through inducible (iNOS) and constitutive NOS (cNOS) is responsible for the reported anti-inflammatory effect of zinc (219). This zinc effect is based on the central role of MTs in affecting NO-mediated changes in labile zinc (203, 220). Indeed, iNOS-derived NO can nitrosate the cysteine thiol groups of MTs and other zinc-containing intracellular proteins, releasing bound zinc and increasing labile zinc (221).

In turn, the increase in intracellular zinc may have a cytoprotective effect by limiting the production of NO through the inhibition of iNOS (222). Spahl et al. (223) demonstrated that iNOS-derived NO causes a transient increase in free zinc concentration in the nucleus that correlates with the translocation of MTs to the nucleus, where they rapidly exchange zinc with zinc-finger transcription factors and regulate gene expression. Indeed, under the influence of NO, zinc released from MTs suppresses the expression of iNOS, through inhibition of NF-κB transactivation (222, 224), whereas it promotes the expression of MTs, which, in turn, can scavenge NO through covalent binding to form S-nitrothiols (223) (**Figure 2B**). In addition, Berendji et al. (225) demonstrated that labile zinc increased in the nucleus after exposure to NO. The local release of zinc appears to be associated with the release of the metal from zinc-containing domains of transcription factors as a result of nitrosylation of zinc thiolate clusters and consequent disruption of these domains, which affects gene expression. These results suggest that the cytotoxicity of excessive and long-term NO exposure, as in inflammation, directly targets zinc clusters in transcription factors that destroy DNA-binding activity (202). Prolonged exposure to NO disrupts zinc homeostasis in pancreatic islet cells and reduces the pool of labile zinc (226). In addition, NO could S-nitrosate the thiols of the MTs and cause dissociation of the zinc from the sulfur ligands of the MTs. As indicated by the reduction in zinc levels, it is conceivable that the zinc released from MTs is not properly handled during nitrosative stress, suggesting that pancreatic islet cells lose the ability to efficiently complex and store zinc under these conditions (226).

On the other hand, NO as well as other free radicals have been shown to play a key role in the dysfunction and damage of pancreatic β-cells in diabetes, in part due to the intrinsic susceptibility of β-cells to OS (227). The role of NO in insulin secretion is less clear and sometimes contradictory. Indeed, physiologically low levels of NO, produced by cNOS, stimulate insulin secretion. In contrast, cytokines induce iNOS expression in islet β-cells in response to inflammatory stimuli, resulting in the production of an excessive amount of NO (228), which is directly involved in cytokine-mediated inhibition of insulin secretion and islet degeneration (229). In this regard, zinc dyshomeostasis induced by excessive amounts of NO could have an important impact on the ability of pancreatic β-cells to release insulin because zinc is involved in the crystallization and storage of insulin in secretory granules (113).

Although the role of NO in regulating adipocyte differentiation is still unclear, a similar mechanism has been proposed to be responsible for the upregulation of adipocyte differentiation leading to the development of obesity. Specifically, abnormal production of NO could trigger intracellular zinc mobilization that positively affects adipocyte differentiation (230). However, the role of isoforms of NOS in this process remains to be elucidated (231, 232).

### 6.2 Zinc muffling and buffering

The short lifetime of zinc signals themselves implies that when cytosolic zinc concentration increases, cells are able to control it, i.e., they generate zinc signals of different amplitudes, minimize cytosolic zinc fluctuations, and eventually restore the equilibrium state of zinc concentration. As for calcium (233), all processes involved in the control of zinc transients are referred to as zinc “muffling”. Together, the buffering and muffling processes not only ensure that the free cytosolic zinc concentration remains low, thereby protecting the cell from the toxic effects of zinc overload but also modulate the availability of zinc ions for binding to proteins that require them. The molecular basis of the buffering and muffling processes is based on the interplay between MTs, and specific zinc-transporting proteins involved in zinc distribution (116).

In this context, in addition to their frequently cited function as zinc buffers, MTs also function as zinc attenuators. Because MT-bound zinc can be mobilized, they are not only zinc scavengers but also able to dynamically release zinc ions to or accept them from other metal-binding proteins and make the metal ion available to other proteins. Although these two functions appear quite different at first glance, they are closely related and depend on the coordination of zinc binding sites on MTs and thiol reactivity. The partial saturation of the zinc-binding sites of MTs in the resting state allows them to bind additional zinc ions in the event of an increased concentration of free zinc (178, 234). Although important, either the binding affinity or even cellular expression of MTs alone is sufficient to account for total cellular zinc buffering while ensuring maintenance of zinc concentration at a critical level and subsequent return to baseline (235). On the other hand, excessive cytosolic zinc buffering capacity prevents zinc fluctuations rapidly extinguishing any zinc signals. In this context, several studies have shown that MTs play a role in controlling the intracellular free zinc pool by zinc muffling. It is based on the ability of MT to attenuate the increase in cytosolic zinc by translocating into cells and supplying zinc to their specific transporters located at the plasma membrane or the membrane of subcellular compartments when the concentration of free zinc is elevated (236, 237). Indeed, in addition to the cytosol, MTs have been found in various cellular compartments such as the nucleus and the intermembrane space of mitochondria (238–240).

As for MTs, the activity of zinc transporters, which transport zinc from the cytosol to the extracellular space or sequester zinc in intracellular compartments, contributes to zinc buffering, allowing the cell to store zinc and rapidly release it when needed temporarily. The zinc buffering capacity of MTs, in conjunction with their translocation within cells and the activity of zinc transporters, are collectively responsible for zinc muffling.

Hitherto, twenty-four mammalian transporters have been described. They belong to two complementary protein families: the Zinc Transporter (ZnT) and the Zrt, Irt-like Protein (ZIP), which in humans are encoded by the Slc30a1-10 and Slc39a1-14 genes, respectively (241). In addition to the plasma membrane, zinc-transporting proteins have been found on ER membranes, in mitochondria, in the Golgi apparatus, intracellular vesicles, and lysosomes; in contrast, the nuclear membrane appears to be devoid of specific zinc transporters (237). Although both protein families share selectivity for zinc binding, ZnT and ZIP transporters move zinc in opposite directions. ZnTs are responsible for removing excess zinc from the cytoplasm and transporting it out of cells or into the lumen of intracellular compartments, whereas ZIPs promote the influx of zinc from the extracellular space or intracellular stores into the cytoplasm. Thus, ZnTs prevent cellular overaccumulation of zinc, whereas ZIPs replenish cytosolic zinc. While most ZIPs are localized at the plasma membrane, most ZnTs are localized in the intracellular compartments, except ZnT1, which is on the cell surface, where it functions as the major pathway of zinc efflux and provides control of metal ion levels (242–244).

In addition to maintaining zinc homeostasis, ZnTs and ZIPs enable the compartmentalization of zinc and play an important role in zinc movement across the compartment membrane in which they are localized (245). They promote zinc entry into the lumens of subcellular compartments, where it is required (for zinc proteins, e.g., zinc-containing enzymes (246); on the other hand, they mediate the local release of zinc in the cell, which is accompanied by zinc transients through which the zinc ion affects gene expression and cell signaling (247). The cooperative regulation of MTs and ZnT transporters, whose expression is tissue- and cell-specific, is essential for zinc homeostasis (35).

The expression and cellular distribution of several physiological mediators regulating MTs and specific ZnT and ZIP proteins are strictly regulated by zinc availability (248, 249). For instance, excessive zinc boost increases ZnT1 surface expression, whereas zinc deficiency causes ZnT1 internalization and degradation (250).

## 7 Zinc transporters and obesity

Because zinc homeostasis is primarily controlled by zinc transporters, a possible explanation for the different zinc levels and distribution in obesity could be changes in the expression and activity of these zinc-regulating proteins. Changes in the expression of zinc transporters may be due, at least in part, to the inflammatory state characteristic of obesity. Indeed, an inverse relationship between inflammatory markers, BMI, and body fat percentage and the expression of various zinc transporters such as ZnT4, ZnT5, ZnT9, ZIP1, ZIP4, and ZIP6 was found in obese women (251). Similarly, the expression of ZnT1 and ZnT5 was upregulated in leukocytes in another zinc intervention study in obese subjects. Most importantly, an increase in the expression of both zinc transporters was positively correlated with zinc intake in both females and males, although it reached a higher statistical significance in females (251). This suggests that changes in expression levels, mainly of ZnT1, are a more sensitive marker of zinc status than circulating zinc concentrations. ZnT1 upregulation was also observed in the blood samples of obese women, followed by ZIP1 (252). Not surprisingly, the expression of ZnT1, the only transporter responsible for the efflux of zinc from cells, is directly controlled by the availability of zinc and its expression decreases with low zinc intake to protect cells from excessive zinc loss (253). Similarly, the upregulation of ZnT5 serves to restore zinc levels in cellular secretory pathways, particularly in the Golgi apparatus, where luminal zinc is loaded onto secreted proteins that require zinc for their catalytic activity (254).

Since the first report on the expression pattern of ZIPs and ZnTs in adipose tissue, it has become apparent that the biology of the different fat depots, namely subcutaneous and visceral fat (VAT), correlate to different expression levels of zinc-transporting proteins (255). Further changes observed in these depots from lean and obese individuals have reinforced the notion that zinc differentially affects lipid metabolism according to metabolic contest, ultimately reflecting the differential expression of zinc transporters.

**Figure 3:**
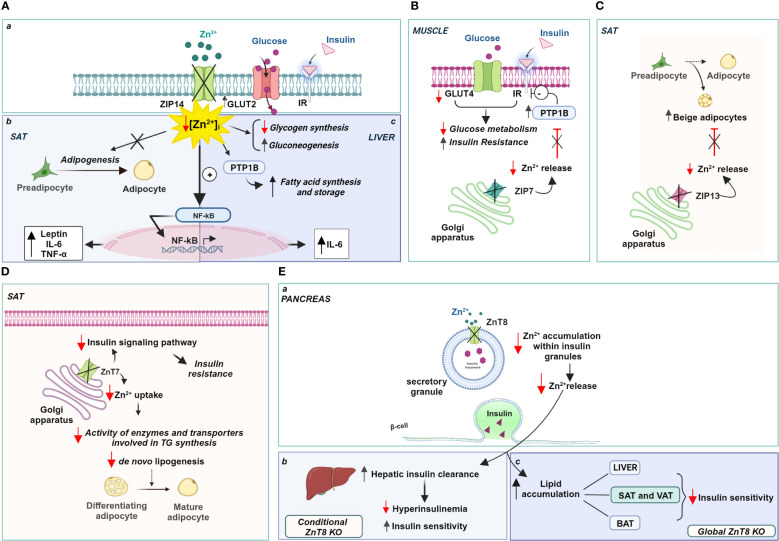
Zinc-dependent pathways in obesity: role of ZIPs and ZnTs. **(A)** Localization (a) and role of Zip14 in the SAT (b) and liver (c); **(B)** Zip7 functional role in the Golgi apparatus of muscle, **(C)** Zip13 and **(D)** ZnT7 functional role in the Golgi apparatus of SAT; **(E)** ZnT8 role in the regulation of insulin release (a) and insulin sensitivity (b, c). Created with BioRender.com.

In this regard, alterations in the expression of zinc transporters were found in the subcutaneous adipose tissue (SAT) of obese patients. In particular, the expression of ZIP14 showed a significant and reversible reduction in these fat depots of obese individuals, which was restored after a period of weight loss (256). Interestingly, ZIP14 expression increased sharply during the early differentiation of preadipocytes into mature adipocytes, suggesting a role for this transporter in adipogenesis and not during lipogenesis (257). It is likely that the localization of ZIP14 to the plasma membrane is responsible for zinc influx into preadipocytes and controls the intracellular zinc increase that regulates the final differentiation of preadipocytes into mature adipocytes. Therefore, reduction or deletion of ZIP14 negatively affects adipose function, impairs late adipocyte differentiation, and promotes the acquisition of a hypertrophic phenotype often associated with insulin resistance. Notably, Troche and coworkers (258) elegantly demonstrated that deletion of ZIP14 alters the metabolism of white adipose tissue (WAT), rendering it insulin insensitive and increases the expression of cytokines such as leptin and IL-6 by disinhibiting NK-kB and JAK2/STAT3 signaling pathways (**Figure 3A** a, b). The reduction of ZIP14 in obese patients negatively correlated with both leptinemia and adipose tissue leptin levels in obesity, as in ZIP14 knockout mice, which exhibited higher levels of leptin. Altogether, knockout of ZIP14 mimics a state of zinc deficiency similar to that observed in obese individuals, and the occurrence of metabolic changes similar to those observed in adipose tissue of obese individuals demonstrates that ZIP14 is critical for controlling zinc availability in metabolism and in inhibiting inflammatory processes. Remarkably, ZIP14 knockout mice showed hyperinsulinemia and body fat accumulation, two major features of type 2 diabetes and obesity (259). Moreover, in obese adipose tissue, ZIP14 downregulation has been associated with the increased expression of several cytokines, such as TNF-a and IL-10 (256).

Similarly, ZIP14 expression has functional significance in hepatocytes under physiological conditions. Up-regulation of ZIP14 is responsible for an increase in liver zinc content and a concomitant decrease in serum zinc, which, in turn, is directly related to zinc transport into hepatocytes. Accordingly, downregulation of ZIP14 and consequent zinc deficiency was observed in a mouse model of alcoholic liver disease (260), Moreover, the lack of ZIP14 upregulation in both IL-6 and iNOS knockout mice clearly suggests that the expression of this zinc transporter is under the control of pro-inflammatory cytokines, namely IL-1 β and IL-6 (261, 262). Interestingly, inflammation-induced increase in zinc levels regulates inflammatory response inhibiting further IL-6 release and interfering with NF-kB activation (262) (**Figure 3A** a, c).

ZIP14 is also involved in regulating hepatic glucose metabolism with opposite effects compared to adipose tissue. The abundance of ZIP14 on the plasma membrane of hepatocytes is strongly regulated by postprandial glucose metabolism. At this stage, higher ZIP14 expression regulated the intensity and duration of insulin action. Indeed, ZIP14 directly controlled insulin signaling through the activation of the two endosomal enzymes, cathepsin D and insulin-degrading enzyme (IDE), which are responsible for dissociating insulin from its receptor (263). ZIP14 KO mice had a higher glucose transport rate and higher glucose concentration in the liver than WT mice, due to a stronger expression of the glucose transporter GLUT2 at the plasma membrane. Interestingly, decreased expression of ZIP14 not only did not affect the insulin sensitivity of hepatocytes but also potentiated hepatic glucose metabolism and promoted glycogen synthesis. Hence, it is conceivable that ZIP14-mediated zinc influx is essential for fine-tuning insulin signaling in hepatocytes and positively regulates glucose metabolism by promoting both glycolysis and gluconeogenesis (263) (**Figure 3A** a, c).

Consistent with the role played by ZIP14 in regulating glucose-lipid metabolism, induction of ZIP14 in hepatocytes also assumes a relevant role in adaptation to ER stress induced by high-fat diet (HFD). Indeed, ZIP14 increased cellular zinc availability and reduces the risk of liver disease (264). Mechanistically, ZIP14 mediated the influx of zinc that inhibits PTP1B activity in the liver and negatively affects the hepatic synthesis of fatty acids and storage of triglycerides during HFD feeding (265). In contrast, the ablation of ZIP14 has a negative effect favoring triglyceride accumulation because PTP1B activity is not inhibited due to the reduced availability of zinc (264) (**Figure 3A** a, c).

Similarly, studies recapitulating high-fat diets indicate the involvement of the zinc transporter ZIP7 in insulin signaling and glucose metabolism in skeletal muscle cells (266). ZIP7 is responsible for the zinc homeostasis of the Golgi apparatus transporting zinc out of the lumen of this subcellular compartment and contributing to an increase in intracytoplasmic zinc concentration (267). Physiologically, the function of ZIP7 is associated with glycemic control through a positive modulation of the molecular components of the insulin signaling pathway. ZIP7 thus supports glucose uptake and its use for glycogen synthesis in skeletal muscle. Interestingly, mice fed a high-fat diet exhibited lower expression of ZIP7, consistent with the onset of insulin resistance in muscle (266) (**Figure 3B**). The exact mechanism by which ZIP7 is involved in insulin resistance and, more importantly, how its zinc transport activity relates to its physiological effects in skeletal muscle requires further investigation, as opposite changes in ZIP7 expression have been found in diabetic cardiomyocytes (268).

Recently, ZIP13, a transporter localized in the Golgi apparatus that mediates zinc transport into the cytoplasm (269), has been linked to the biogenesis of the so-called “beige adipocytes”. They derive from the browning of white adipose cells in visceral tissue and display functional properties intermediate between brown (BAT) and white adipocytes (270). Indeed, beige adipocytes accumulate lipids like white adipocytes and produce heat like brown adipocytes, improving insulin sensitivity and glucose metabolism. They are a potential new therapeutic target for treating metabolic disorders such as obesity, which is known to be associated with decreased thermogenesis. In this context, ZIP13 has attracted interest because it is highly expressed in pancreatic β-cells and the gene encoding this zinc transporter is one of the genes involved in glucose homeostasis during fasting (271). A loss-of-function mutation in the ZIP13 gene has been associated with Ehlers-Danlos syndrome, which is characterized by decreased white adipose tissue mass, among other pathological manifestations (269). Interestingly, ZIP13-deficient mice show increased biogenesis of beige adipocytes due to accelerated differentiation of preadipocytes into beige cells, suggesting that ZIP13 physiologically acts as a negative regulator of adipocyte browning. ZIP13 likely provides zinc ions for modulating the activity of enzymes responsible for degradation of the key components of the adipocyte browning process (272) (**Figure 3C**).

Further evidence supporting the role of zinc transporters in adipose tissue metabolism was provided by ZnT7 KO mice. ZnT7 is ubiquitously expressed and localized in the Golgi apparatus, promoting zinc influx into this subcellular compartment’s lumen (273). Huang and colleagues (274) have observed that ZnT7 KO mice display a lower body fat percentage than their counterparts WT. These results were subsequently confirmed by Tepaamorndech and coworkers (275), which elegantly demonstrated that adipose tissue is the only tissue affected by ablation of ZnT7, with no relevant differences in other body tissues. Accordingly, since the synthesis and release of leptin are directly dependent on the extent of fat mass, in parallel with a reduction in fat depots, ZnT7 KO mice exhibit decreased circulating leptin levels (276). The main effect on lipid metabolism observed in knockout mice can be attributed to the reduced availability of zinc in the Golgi apparatus which is required for the proper activity of enzymes and transporters involved in triglyceride synthesis.

In addition, ZnT7 is differentially expressed in subcutaneous and visceral fat pads, with a higher expression of ZnT7 in SAT. As a result, the main effects of ZnT7 deficiency have been observed in SAT fat cells, showing a size reduction, probably due to reduced lipid accumulation. Importantly, ZnT7 expression within SAT is controlled and occurs only when adipocyte lipogenesis and not differentiation is induced (275). Genetic ablation of ZnT7 impaired the ability of fat cells to synthesize lipids resulting in insulin insensitivity and glucose intolerance in SAT adipocytes (**Figure 3D**). Reduction of ZnT7 expression impaired insulin signaling pathway activity and decreased glucose uptake by quantitatively reducing insulin-stimulated activation of Akt. These changes decrease the glycolysis rate and thus the availability of metabolic intermediates necessary for fatty acid production, altering lipogenesis within SAT. From a whole-body perspective, the reduced sensitivity of SAT to insulin action results in a significantly poorer ability of the body to store excess glucose and lipids. In light of these findings, it can be argued that the higher expression of ZnT7 in SAT appears to confer unique properties to this fat depot that predispose it to the accumulation of excess fat, reducing the risk of developing metabolic abnormalities associated with obesity (277). It is worth noting that ZnT7 deficiency is associated with a negative zinc status in the body that cannot be corrected by zinc supplementation. This suggests that ZnT7 expression is not dependent on and cannot be modulated by dietary zinc intake.

Significant alterations in body fat homeostasis and glucose tolerance have been observed in ZnT8 knockout mouse strains. ZnT8 was first identified on insulin granules in pancreatic β-cells, where ZnT8 operates by accumulating cytoplasmic zinc inside the granules (278). The identification of polymorphisms in the gene encoding ZnT8 associated with type 2 diabetes in nonobese individuals (279, 280) and the presence of autoantibodies to ZnT8 in patients with type 1 diabetes has generated considerable interest in the specific role of this transporter in obesity. However, it has been shown that specific deletion of ZnT8 in pancreatic β-cells does not increase the risk of developing obesity but, on the contrary, protects against insulin resistance induced by a high-fat diet (281). Indeed, ZnT8 deficiency prevents the hyperinsulinemia often observed with high fat intake and maintains insulin sensitivity. It is conceivable that reduced zinc accumulation in insulin granules due to specific ZnT8 deficiency of β-cells does not lead to hyperinsulinemia because hepatic clearance of pancreatic hormone is increased (282). Zinc contained in insulin granules and released along with insulin reduces hepatic degradation of the hormone by inhibiting its endocytosis and subsequent degradation, thus ensuring proper insulin delivery to target tissues. Hence, the loss of this endocrine effect of zinc in ZnT8 KO mice affects the rate of hepatic insulin excretion (**Figure 3E** a, b).

The scenario changes dramatically in global ZnT8 knockout mice exhibiting severe insulin resistance and obesity. Indeed, Mao and colleagues (283) found that global KO mice, in contrast to conditional β-cell ZnT8 KO mice, exhibited adipocyte hypertrophy due to lipid accumulation in all major white adipose depots (VAT and SAT), accumulation of lipids in the liver, and increased expression of genes related to fatty acid synthesis and uptake. Notably, BAT also accumulated more lipids upon ZnT8 ablation, although the expression of genes controlling energy expenditure through heat production was not affected. In this contest, ZnT8 deficiency combined with a high-fat diet significantly exacerbated the effects caused by the absence of this zinc store alone and contributes to increased obesity (283) (**Figure 3E** a, c).

## 8 Conclusions

Zinc abnormalities are considered a common feature of obesity, and zinc supplementation strategies are attracting considerable interest as a potential strategy to improve body weight management, inflammatory biomarkers, and insulin resistance in obese individuals. However, zinc supplementation does not produce consistent results and is not always successful. Zinc deficiency likely exacerbates the general state of micronutrient deficiency characteristic of obese individuals. At the same time, the diversity of mechanisms affected by zinc deficiency underscores the indispensable role that zinc plays physiologically and, more importantly, in pathological conditions.

In recent years, new insights have been gained into the function of various zinc transporters. The strict interplay between MTs and members of the ZIPs and ZnTs families is critical for zinc buffering and muffling. In addition, there is a close functional relationship with redox metabolism. Changes in zinc buffering and muffling capacity assume a central role under physiological conditions, but even more so under conditions characterized by oxidative stress, as in obesity. We are beginning to determine how individual zinc transporters may be involved in obesity. However, a more comprehensive view of the full spectrum of alterations in zinc homeostasis can improve the understanding of the mechanisms underlying obesity and its associated comorbidities and develop novel therapeutic strategies aimed at reducing the impact of obesity.

## Author contributions

CF: Conceptualization, Writing – original draft, Writing – review & editing. LC: Writing – review & editing, Conceptualization, Funding acquisition, Writing – original draft.
